# Sleep patterns and habits in high school students in Iran

**DOI:** 10.1186/1744-859X-7-5

**Published:** 2008-03-13

**Authors:** Ahmad Ghanizadeh, Mohsen Kianpoor, Mehdi Rezaei, Hadi Rezaei, Rozita Moini, kamran Aghakhani, Jamshid Ahmadi, Seyed Reza Moeini

**Affiliations:** 1 Department of Psychiatry, Shiraz University of Medical Sciences, Shiraz, Iran; 2 Research Center for Psychiatry and Behavioral Sciences, Shiraz University of Medical Sciences, Shiraz, Iran; 3 Shiraz University of Medical Sciences, Shiraz, Iran; 4 Department of Forensic Medicine, Iran University of Medical Sciences, Tehran, Iran; 5 Hazrat Fatemeh Heart Center, Shiraz, Iran

## Abstract

**Background:**

Sleep patterns and habits in high school students in Iran have not been well studied to date. This paper aims to re-address this balance and analyse sleep patterns and habits in Iranian children of high school age.

**Methods:**

The subjects were 1,420 high school students randomly selected by stratified cluster sampling. This was a self-report study using a questionnaire which included items about usual sleep/wake behaviours over the previous month, such as sleep schedule, falling asleep in class, difficulty falling asleep, tiredness or sleepiness during the day, difficulty getting up in the morning, nightmares, and taking sleeping pills.

**Results:**

The mean duration of night sleep was 7.7 h, with no difference between girls, boys, and school year (grade). The mean time of waking in the morning was not different between genders. About 9.9% of the girls and 4.6% of the boys perceived their quality of sleep as being bad, and 58% of them reported sleepiness during the day. About 4.2% of the subjects had used medication to enhance sleep. The time of going to bed was associated with grade level and gender. Sleep latency was not associated with gender and grade leve, l and 1.4% experienced bruxism more than four times a week.

**Conclusion:**

Our results are in contrast with that of previous studies that concluded sleep duration is shorter in Asia than in Europe, that boys woke-up significantly later than girls, and that the frequency of sleep latency category was associated with gender and grade level. The magnitude of the daytime sleepiness, daytime sleepiness during classes, sleep latency, and incidences of waking up at night represent major public health concerns for Iran.

## Background

Sleep patterns in adolescence vary with age, ethnic group, lifestyle issues, cultural beliefs, family values and socio-cultural influences. Adolescent sleep patterns effect the way sleep problems are defined. Therefore, considering socio-cultural differences in sleep patterns is an important factor for the assessment of sleep.

### Sleep habits

There is a great difference between countries in duration of sleep time among students. The duration was 8 h 30 min for the Finish [1], more than 9 h for the Swiss [1], 6 h 20 min for the Japanese [2], 4.8 to 6.2 h for Koreans [3], 5.4 h in a multinational study [4], 7.5 h for the Chinese [5], and 7 to 8 h for American students [6]. Additionally, sleep duration appears not to be gender-related [3,7].

A study on sleep/wake patterns among Korean teenagers showed that as the school year (grade) level increased, students went to bed later, woke up earlier, and slept less. Boys awoke significantly later than girls [3].

About 61% of the Korean students had not woken up at night during the previous 2 weeks, and the frequency increased across grades 9 to 12 from 51.3% to 56.3%, 63.0%, and 79.0%, respectively [3].

Sleep latency was longer in girls than boys [3]. Daytime sleepiness was reported to be 19.9% [6], increasing with age in adolescents [1]. Male adolescents fell asleep later than girls [4]. About 14.9% of 12- to 18-year-old adolescents in China reported difficulty falling asleep on at least 4 nights in the past month [8].

### Sleep-related symptoms

A multinational study on adolescent sleep reported early morning waking in 10.5%, occurrence of nightmares in 4.2%, and sleepwalking in 5.0% [6]. Breathing disorders (e.g. obstructive sleep apnoea) and primary snoring occurred in as many as 11% of the paediatric patients [9].

The prevalence of daytime sleepiness in teenagers significantly increased from grades 9 to 12 from 2.1% to 3.2%, 10.7%, and 13.9%, respectively and the prevalence was more in girls than boys. The rate of falling asleep three times or more per week in class was 18%. The rates significantly increased with grade at 4.0%, 5.5%, 11.6%, and 24.9%, respectively [3].

About 4% of Italian students had used medication to help themselves sleep at least once during the last 6 months and 1.3% had regularly taken sleeping pills. Girls were more prevalent users of sleep medication than boys [10].

Adolescents in Iran are from a generation whose parents have been involved in a long-lasting and devastating war and now the adolescents are directly and indirectly facing the many consequences of that war. In addition, there is no adequate time allocation for nap taking and eating breakfast or lunch at school. These factors do not seem to be present in the Western world.

This study surveys the self-reported night- and daytime sleep habits of adolescents in Shiraz, one of the largest cities in Iran. Most of the research in this field to date has been conducted in the Western world and, to our knowledge, there is no other study that has reported sleep patterns and sleep quality among Iranian adolescents, who are from a non-Western culture.

## Methods

### Subjects

In order to take a sample that was representative of the group of subjects that we intended to study, eight all-male high schools and eight all-female high schools were randomly selected from strata defined by age, grade and sex. About 350 students (about 175 girls and 175 boys) were selected from each age group based upon stratified cluster sampling. A total of 1,500 students were selected, with 1,420 (94.6%) participating in the study. All subjects chosen were from Shiraz, one of the largest cities in Iran. All the students attending school on the day of the survey were invited to participate. The participating subjects were 1,420 high school students, of which 696 were boys (49.0%). The participants' mean age was 16.4 years and the age range was 15 to 18 years.

### Study instrument

The questionnaire used was a comprehensive instrument including items about usual sleep/wake behaviours over the previous month. It asked about the schedule of sleep, falling asleep in class, difficulty falling asleep, tiredness or sleepiness during the day, and difficulty getting up in the morning. It also asked about occurrence of nightmares (dreams with content that the sleeper finds disturbing), leg movement (periodic episodes of repetitive and stereotyped limb movements in sleep), sleep talking (utterance of speech or sounds during sleep), sleep walking (a series of complex behaviours that result in movement during sleep), bruxism (grinding of the teeth during sleep), use of sleeping pills and whether the subject was in the habit of drinking coffee late in the evening [11]. "Sleepiness during class" is different to "falling asleep in class". The first means that the subject is in a sleepy state, but is not actually asleep. The second one refers to actual sleeping. The instrument contained questions to which the subjects responded using a 1- to 5-point ordinal scale. It assessed the frequency of the respective problems during the week (1 = never, 5 = almost every day or night). Four questions required categorical answers and assessed what time the subject went to bed, the length of time needed to fall asleep, length of daytime naps, and the number of nocturnal awakenings. Sleep quality was rated from excellent (1) to very poor (5). Most questions concerned the whole week. The questionnaire was translated into Farsi and was back-translated to ensure that the questions retained the same meaning.

### Procedure

After a preliminary study to evaluate if the questions were understood as expected by the students, the final version was used in the current study. The questionnaire was administered by trained medical students in the period from 20 January to 20 February 2005 in high school classes during morning school hours. Participation of the students in the study was voluntary, and informed consent was taken from the participants, teachers and principals and the educational administrative headquarters in Shiraz. The study protocol was approved by the Ethics Committee of Shiraz University of Medical Sciences.

### Statistical analysis

The data were analyzed using SPSS software for Windows (SPSS Inc., Chicago, IL, USA). The Student's t test and Chi-square tests were used to examine group differences for discrete data. Analysis of variance (ANOVA) was used to examine gender differences and grade effects on the variables. The level of significance was considered at p = 0.05.

## Results

### Sleep habits

#### Time of going to bed

About 14.2% of the subjects went to bed before 10 pm and 19.6% went to bed after midnight. The time of going to bed (TGT) was associated with grade level (Chi-squared = 37.03, df = 12, p < 0.001). Adolescents in grade 11 and 12 went to bed later than those who were in grades 9 and 10. In addition, TGT was associated with gender (Chi-squared = 30.05, df = 4, p < 0.001) and girls usually went to bed earlier than boys.

#### Sleep latency

The question on sleep latency of students showed the following distribution of sleep latency categories: less than 6 min in 12.2%; > 5 and < 11 min in 35.8%; > 10 and < 31 min in 35.4%; > 30 min in 16.7%. Sleep latency was not associated with gender (Chi-squared = 7.3, df = 3, p = 0.06) or grade level (Chi-squared = 2.24, df = 9, p = 0.98).

#### Waking at night

The question about waking at night showed the following distribution categories: never in 44.6%, 1 to 2 times in 47.5%, 3 to 4 times in 5.9%, 5 to 6 times 1.5%, and more than 6 times in 0.6%. It was associated with gender and the frequency in girls was higher than that in boys (Chi-squared = 19.9, df = 4, p < 0.001). In addition, it was associated with grade level (Chi-squared = 21.9, df = 12, p < 0.03).

#### Morning waking time

The mean morning waking time was 07.17 am. The time of waking in the morning in girls was 07.14 (SD = 1.3) and in boys it was 07.19 (SD = 1.3). There was no difference between genders (t = 0.66, df = 1,257, p = 0.5). The mean waking time was different between grade level (F_3,1255 _= 17.2, p < 0.05). The mean morning waking time for 12th grade adolescents was 07.17 (SD = 1.2) while it was 06.57 (SD = 1.3) for the 9th grade subjects.

#### Sleep duration

The mean duration of night sleep was 7.7 h (SD = 1.3). The mean duration was 7.70 h (SD = 1.4) in girls and 7.79 h (SD = 1.3) in boys. There was no difference between girls and boys (t = 1.29, df = 1,329, p = 0.19). The mean durations at different grade levels (grades 9, 10, 11, 12) were 7.63, 7.86, 7.83, 7.65 h, respectively (F_3,1327 _= 4.7, p = 0.057). However, sleep duration decreased from grades 10 to 12.

### Sleep-related symptoms

#### Sleep quality

Sleep quality at night showed the following distribution categories: excellent in 25.6%, good in 41.9%, satisfactory in 25.1%, poor in 5.6%, and very poor in 1.8%. It was not associated with grade level (Chi-squared = 4.2, df = 12, p = 0.97). The quality of sleep was not correlated with age. About 10% of the girls and 4.6% of the boys perceived their quality of sleep as being bad (Chi-squared = 14.9, df = 1, p < 0.001).

#### Daytime sleepiness

About 42% of the students reported that they never or rarely experienced sleepiness during the day. There was no association between daytime sleepiness category and grade level (Chi-squared = 18.2, df = 12, p = 0.10). The frequency of daytime sleepiness in boys was more common than in girls (Chi-squared = 30.8, df = 4, p < 0.001).

#### Falling asleep in school

About 32% of the students never experienced falling asleep in school and 28.9% experienced it less than once a week. Girls experienced this less than boys (Chi-squared = 21.4, df = 4, p < 0.001). The rate of falling asleep was not associated with grade level (Chi-squared = 13.1, df = 12, p = 0.35).

#### Intake of sleeping aids

About 95.8% of the study subjects never used medication to enhance sleep. The rate of medication use was not different between genders (Chi-squared = 6.7, df = 4, p = 0.15). In addition, it was not associated with grade level (Chi-squared = 9.1, df = 12, p = 0.68). About 54.3% of the students drank coffee one or more times per week in the evening and it was associated with onset of sleeping difficulties (Chi-squared = 38.7, df = 16, p < 0.001).

**Figure 1:**
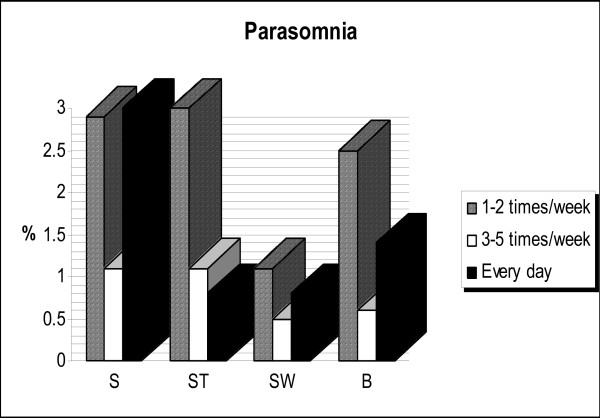
The prevalence of parasomnias such as snoring (S), sleep-talking (ST), sleepwalking (SW), and sleep bruxism (B).

**Figure 2:**
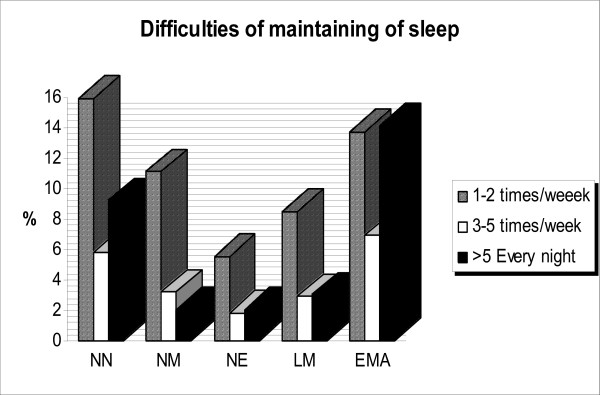
**The prevalence of sleep disturbance symptoms associated with difficulties maintaining sleep:** waking up due to noise at night (NN); waking up because of nightmares (NM); waking up because of nocturnal eating habits (NE); leg movements or disagreeable symptoms (LM); and early morning awakening (EMA).

About 91% of the students never experienced bruxism and 1.4% of them experienced it more than four times per week (Figure 1). Bruxism was not related to either gender or grade level (Chi-squared = 3.1, df = 4, p < 0.5; Chi-squared = 9.7, df = 12, p < 0.6, respectively). The frequencies of nightmares, nocturnal eating habits, leg movement and other items are shown in Figure 2. Occurrence of nightmares was related to grade but not to gender (Chi-squared = 24.7, df = 12, p < 0.01; Chi-squared = 8.7, df = 4, p < 0.06, respectively). Gender and grade were not associated with nocturnal eating habits (Chi-squared = 9.3, df = 4, p < 0.5; Chi-squared = 18.7, df = 12, p < 0.09, respectively).

## Discussion

Comparison between different studies is not an easy task because there is much variability in operational definitions and sample populations, and different measures are used to evaluate sleep. In addition, cultural differences and different lifestyle habits may explain such differences, and the differences may also be due to different secondary school classification. For example, in Italy, 18 year-old adolescents are still attending high school and living with their families while in USA they are already attending college.

### Sleep habits

The mean duration of night sleep was 7.7 h, being higher than that of Korean teenagers at 5.4 h [3]. Also, it was more than Japanese adolescents [2]. However, it was more similar to American adolescents at 7–8 h [4,6]. Our result is contrary to those of previous studies that concluded sleep duration was shorter in Asia than in Europe [12,13]. There is a lot of emphasis on education during high school years in Iran, especially in order to be able to pass the national university entrance examination that is taken at the end of the high school period. Despite the expectation that students should shorten their sleep to study harder in all grade levels of high school, it seems that they usually sleep longer than in Korea and Japan.

Adolescents reported going to bed later as they got older. Studies from other countries such as Canada [14], Australia [15], and Brazil [16] reported similar findings. This might be related to less parental influence, more use of the computer, or on increases in the homework workload.

The mean waking time in the morning was 07.17 am and the time of waking in the morning was not different between genders and the mean was not different between grade levels. This is in contrast with findings from Korea, where boys woke significantly later than girls and the increase of grade level was associated with earlier waking [3]. The Korean study assumed that these differences might result from differences in the time needed to prepare for school and/or family responsibilities. In Iran, female students wear a special uniform including a scarf and usually do not put on make-up. Therefore, preparation is faster and easier and they do not have to wake up earlier. Finally, the absence of significant gender difference in waking time might be a distinct characteristic of Iranian adolescents. It is important to note that school start time is not different for boys and girls and there are no co-educational schools (mixed sex) for adolescent boys and girls in Iran.

Sleep latency is common and the frequency of sleep latency categories was not associated with gender or grade level. This is not in accordance with the results of some previous studies [3,17]. Another previous study reported that difficulty in falling asleep is the most common complaint among adolescents, and there is a trend indicating that longer sleep latency is more frequent in girls than boys [18].

About 44.6% of the students had not woken up at night during the previous 4 weeks, and the frequency increased with grade (grade 9, 10, 11, 12, from 40% to 40.2%, 51.1%, and 47%, respectively). These findings are very similar to the results of the Korean study [3].

#### Sleep-related symptoms

More than half of the students reported experiencing sleepiness during the day. There was no association between daytime sleepiness category and grade level. The rate of daytime sleepiness in boys was more common than girls, being contrary to the results of another Asian study [3].

About 32% of the students never experienced daytime sleepiness in school and 28.9% experienced it less than once a week. The rest of the subjects frequently experienced it. This shows that daytime sleepiness is a very common problem among adolescents in Iran.

About 95.8% of the adolescents never used medication to enhance sleep. This rate is very similar to that of the 4% reported from Italy [10]. Therefore, the use of medications to enhance sleep was not very common among Iranian adolescents.

The magnitude of daytime sleepiness (DS), daytime sleepiness during classes (DSS), sleep latency, waking at night, tiredness in the morning, early morning waking, and night-time snoring represent major public health concerns with respect to sleep patterns in Iran. This study highlights the need for child and adolescent psychiatrists to enquire about sleep problems in this age group, since sleep disturbances in adolescents are frequent.

This study focused only on self-reported questionnaires, and procedures such as electroencephalography or actigraphy were not used. However, other studies on adolescent sleep habits, the results of which were compared with ours, were also self-report studies. Additionally, to date those procedures have not been regularly used in community-based epidemiological studies. Our survey was a cross-sectional study based only on the previous 4 weeks, while the Korean study was based on the previous 2 weeks. Further studies that separate weekday and weekend data are recommended.

## Competing interests

The author(s) declare that they have no competing interests.

## Authors' contributions

AG developed the concept of the study and was the principal investigator and designer and also carried out statistical analysis, interpretation, and writing of the paper. MK participated in interpretation and revision of the paper. MR and HR participated in gathering of the data, statistical analysis, and contributed to the interpretation of the results. RM participated in statistical analysis, interpretation, writing of the paper. KA contributed to the revision of the paper. JA participated in interpretation of results and revision of the paper. SRM participated in interpretation of results and revision of the paper. All authors read and approved the final manuscript.
